# Where are we with unintended effects in genome editing applications from DNA to phenotype: focus on plant applications

**DOI:** 10.1007/s11248-019-00146-1

**Published:** 2019-07-18

**Authors:** Marie-Bérengère Troadec, Jean-Christophe Pagès, on behalf of the Scientific Committee of the High Council for biotechnology, France

**Affiliations:** 1 Univ Brest, Inserm, EFS, UMR 1078, GGB, F-29200 Brest, France; 2 CHRU Brest, service de génétique, UF de cytogénétique, Brest, France; 3 Service de Biochimie et Biologie Moléculaire, Université François Rabelais de Tours, Tours, France; 4 INSERM, UMR 1016, Institut Cochin de Génétique Moléculaire, Paris, France; 5 INSERM U1031 STROMALab, Université Paul Sabatier Toulouse 3, Toulouse, France; 6 IFB Purpan, Service de biologie cellulaire, Toulouse, France; 7 Scientific Committee of the High Council for biotechnology, Paris, France

**Keywords:** Off-target, Genome editing, Plant, Unintended effects

## Abstract

Agriculture has benefited from various conventional techniques for plant breeding, including chemical- or radiation-induced mutagenesis, and to some extent from transgenesis. Genome editing techniques are likely to allow straightforward, cost-effective and efficient gene-specific modifications for identified genetic traits associated to agronomic interest. As for previous plant breeding techniques, genome editing techniques need an appraisal for unintended effects. Hence, an evaluation of potential specific risks associated with genome editing must be considered. The Scientific Committee of the High Council for biotechnology (HCB), using a broad theoretical and literature-based approach, identified three categories of points to consider in terms of hazards in health and environment, as compared to conventional breeding: (1) technical unintended effects related to effector persistence as well as risks associated with off-target modifications or other unintended genome modifications, (2) risks arising from the desired trait and its novelty in the plant, and (3) risks associated with the potential modification of plant breeding practices, owing to efficacy and technical ease-of-use of genome editing (acceleration), be it for single traits or for combined modifications (multiplex genome editing). Due to novelty, HCB also envisions the need for specific risk assessment and management.

## Introduction

Agriculture has benefited from conventional plant breeding techniques. To increase genetic variability, which supports discovery of traits of agronomic interest, breeders have historically used several techniques, including chemical- or radiation-induced mutagenesis, and transgenesis. Recent genome editing techniques (such as CRISPR/Cas9) were shown to allow straightforward efficient gene-specific modifications in plants of agronomic interest. However, as for all breeding techniques, there is a need to evaluate whether these techniques are associated with unintended effects. Here, the questions of the specificity and of the consequence of the ease-of-use of the genome editing techniques focus the attention.

### Genome editing relative to other genome modification techniques

**Fig. 1:**
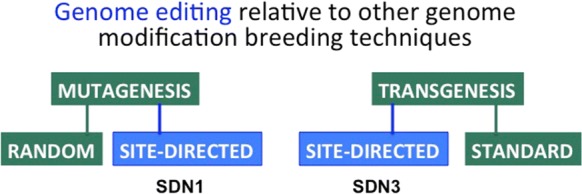
Genome editing relative to other genome modification breeding techniques. Genome editing can generate site-directed modifications that could be compared to mutagenesis or to transgenesis. *SDN* site-directed nuclease. Type of genome editing modifications (blue). Other types of genome modification (light green). (Color figure online)

Genome editing refers to techniques by which specifically designed and engineered molecules promote, insert, replace, or remove DNA within a plant genome with a high degree of specificity. It is first important to indicate how genome edition could be compared to the other traditional/conventional genome modification techniques (Fig. 1). Conventional mutagenesis is achieved through a random process affecting the whole plant genome at multiple genetic loci by physical (e.g.: gamma irradiation), chemical (e.g.: base-analog, alkylating agents, intercalating agent) or biological (e.g.: transposon-mediated) DNA mutagenesis. Those conventional techniques either lack specificity or present low specificity to target sites, which implies a secondary process of backcrosses and filtering to select and enrich the trait of interest in the plant. Transgenesis aims at integrating a DNA fragment encoding for a gene characterized for its agronomic interest into the plant genome. Up to now, this process of integration was unguided which was part of the rationale to enforce a GMO regulation in the European Commission (EC). Petitioners were due to follow a series of directive or regulation defining risk assessment, and other requirements to build the dossier submitted to obtain an authorization for deliberate release of the GM plant in the EC.

The different genome editing techniques were designed to complete or replace either conventional mutagenesis, Site-Directed Nuclease (SDN1, SDN2), or part of transgenesis, Site-Directed Nuclease (SDN3). In all cases, the key characteristic of genome edition is that the modification is site-directed aiming at specificity. It implies that the genome of the targeted plant is at least partially known, and, in the case of SDN1/2, that the function of the targeted gene is defined, while the allele encoding the desired phenotypic trait should be characterized. This constrains the use of the SDN techniques and explains why, at the laboratory level, random mutagenesis remains relevant in the process of trait characterization. Hence, the main rationale for using SDN1/2 as compared to conventional mutagenesis is to facilitate and to accelerate the introduction of a desired trait in varieties by reducing the need for backcrosses.

### The intended effect of genome edition is to achieve site-directed gene modification of heritable characteristics

**Fig. 2:**
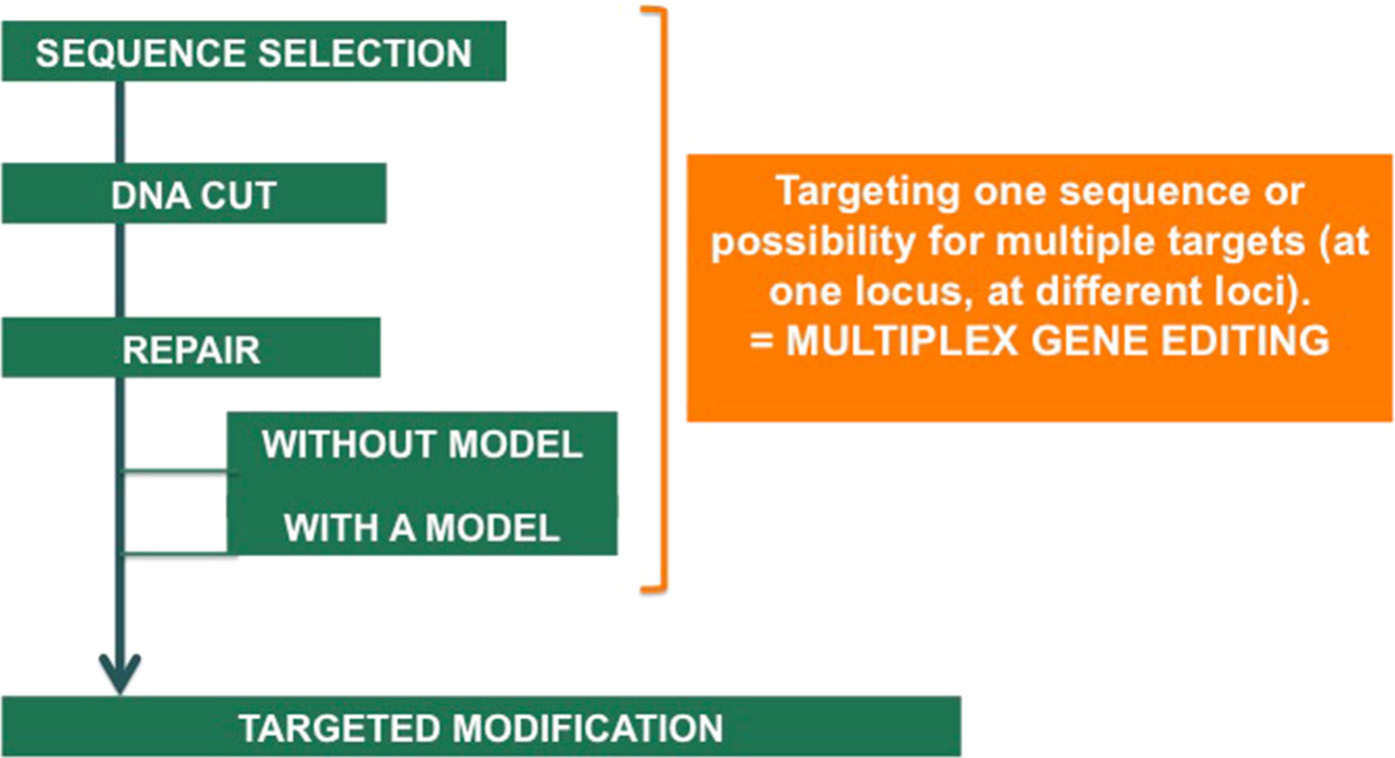
Genome editing, molecular process. Genome edition results from a multi-step process leading to heritable and targeted genome modification. A specific feature of genome editing techniques resulting from its ease-of-use is the opportunity to target multiple genomic sites at once: multiplex gene edition or multiplexing

**Fig. 3:**
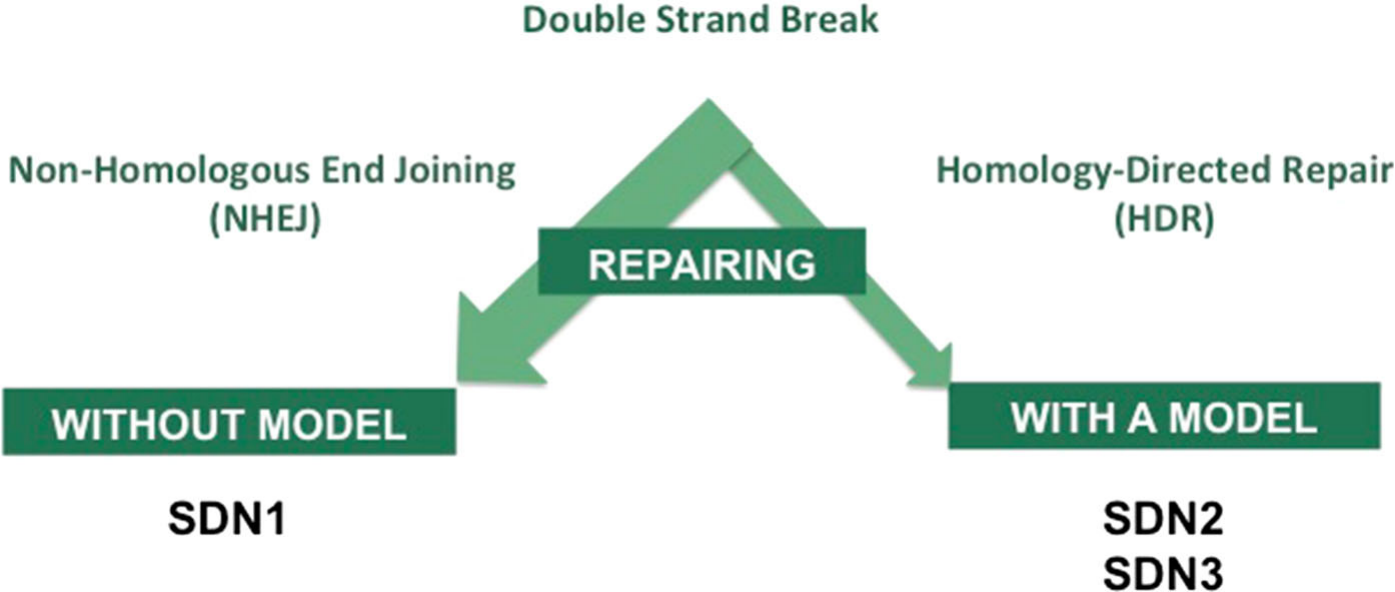
Types of physiological mechanisms for double-strand break repair during genome edition. The non-homologuous end joining (NHEJ) is the main double-strand break repair mechanism, while homology-directed repair (HDR) occurs at low frequency and requires a template. *SDN* site-directed nuclease

Following the choice of the agronomic trait, three molecular steps support genome edition: (1) the targeting of the desired sequence, (2) the DNA-cut at the selected sequence, and (3) the induction of the DNA-repair process (Fig. 2). For each technique, continuous improvement of the specificity is reported for the targeting, ensured for instance by computer-based guide RNA design, and of the cutting steps, for instance using various and variant Cas nucleases. According to the objective sought, either SDN1/2 or 3, the repairing step can involve two different physiological mechanisms (Fig. 3). Technically and up to now, non-homologous end joining (NHEJ) is the easiest mode of repair activated for genome edition. It promotes double-strand-breaks-repair. A selection process is needed to detect the organisms harboring small insertion or small deletion, and leading to the desired gene inactivation supporting the sought agronomic trait. This repair step produces various molecular products; this process denominated SDN1. Alternatively, the repair step can be achieved by soliciting the homology-directed repair mechanism (HDR). This technical step is not trivial, notably because a DNA template/model needs to be delivered within the target cell. Here, the objective is site-directed specific genetic conversion of the target sequence. This repair step guided by a template could generate SDN2 and SDN3 (Fig. 3). It is important to stress that template-guided repair in somatic-derived tissues is outcompeted by NHEJ, and hence remains challenging (Gao 2018). This implies that selection and characterization of the products remain an important step.

In a risk assessment perspective, it is also important to consider genome editing technique as a tool allowing multiplex gene editing. Multiple genomic sites can be targeted at once, either at one locus (i.e. several changes within one gene) or at multiple loci (Fig. 2). This exclusive characteristic constitutes a mean for targeting gene family or metabolic pathways. Also, by mean of in vitro evolution, it is possible to envision a gene conversion process leading to the expression of new functions within an organism. We will discuss these points in the following sections.

## Problematics and discussion

Genome editing is expected to produce intended effects (site-directed and specific modifications), it is however legitimate to question possible unintended effects and propose proportional evaluation if needed. Consequently, setting a list of possible mechanisms related to the techniques and leading to undesirable effects would help. Based on this theoretical list we could assess the occurrence of such deviation, the safety considerations raised, and, if relevant, the management measures which could be proposed to prevent, control or limit potential unintended effects.

### Unintended effects: from the genome, the cell and plant phenotypes to the field scale

**Fig. 4:**
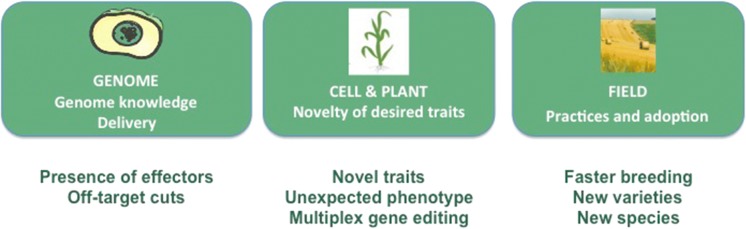
The three scales and the type of unintended effects to consider in genome edition

When thinking about potential unintended effects, we need to consider the different levels at which they would arise and affect organisms (Fig. 4). First, at the level of the genome referring to the type of risks inherent to the genome editing technique itself. Second, at the phenotypic scale from the cell to the plant and the desired trait. And finally, at the scale of the field and agricultural practices, here related to the trait and the range of adoption of the varieties in the environment.

### Genome modification: unintended effects inherent to the genome editing technique itself

It is possible to distinguish two types of unintended effects associated with the technique itself, those associated with (1) the molecular tools and the possible persistence of effectors, and (2) an off-target activity of these tools.

### A (1) unintended effects associated with effector persistence

Genome editing is performed by molecular tools which could be expressed from “effectors”. The active molecules are specialized enzymes acting as duplex (for Zinc Finger Nuclease, ZFN or TALEN) or associated to a guide RNA (gRNA) in the case of CRISPR/Cas nucleases. The stable or transient presence of the effectors depends on the molecular constitution of the tools used to express the enzymes. Depending on technical opportunities or choice, an identical modification could be obtained by expressing the editing activity from: a DNA, stably integrated into the target genome, a DNA transiently introduced into the target cell, a RNA encoding the enzyme and the guide, or the enzyme itself as nuclease-protein and the gRNA. Each of these molecules need to be introduced within the target organism by various delivery systems.

**Fig. 5:**
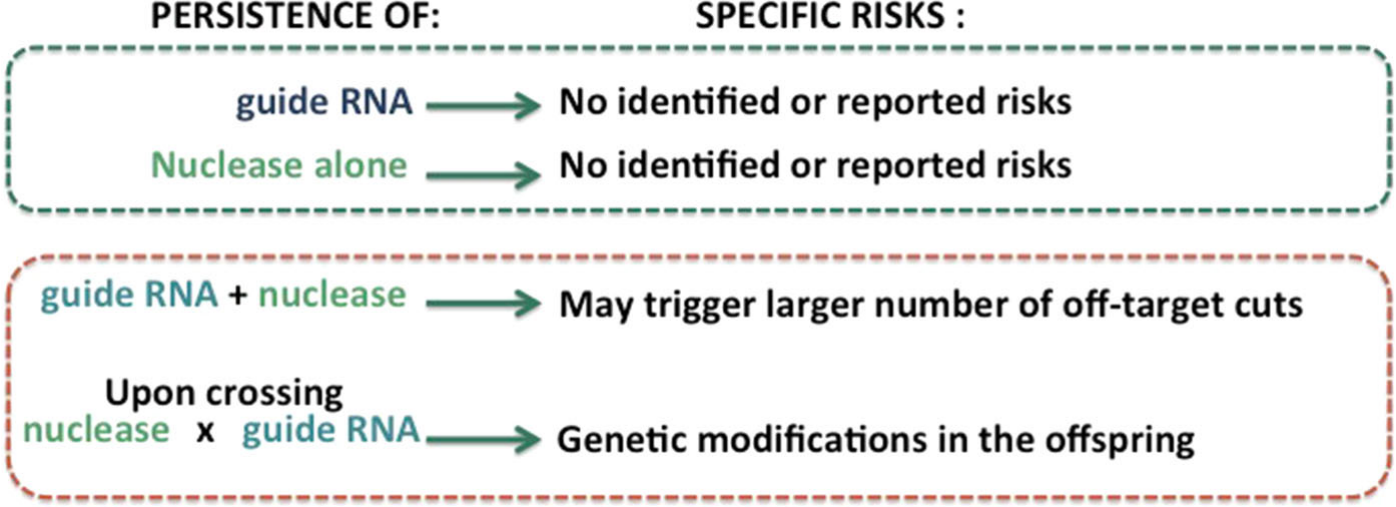
Potential risks resulting from the presence of the effectors. Effector refers to molecular tools that are transiently or stably introduced within the cell for genome edition. Effectors are the specialized enzymes (e.g. Cas9 nuclease) and guide RNA (gRNA) or the genetic sequences encoding these molecules

While the consideration supporting the technical choice made by growers will not be discussed here, it affects the consideration on unintended events. Persistence of either gRNA or nuclease alone does not seem to be associated with any risks, since editing activity relies on the presence of both. However, persistence of integrated DNA encoding these molecules in the target organism qualifies the organism as a GMO. Moreover, persistence of gRNA together with a nuclease maintains activity and could induce a certain number of off-target cuts. Also, crossing of plants containing functional effectors (for example, a plant containing Cas9 nuclease with a plant containing gRNA) is expected to result in genetic modifications in offspring (Fig. 5). The Scientific Committee of HCB recommends verifying (by PCR, RT-PCR, sequencing…) that effectors remain absent in the final plant. If effectors are genetically present and stably transmitted, the plant is transgenic. In this case, adequate regulation such as European GMO Directive 2001/18/EC applies. In the absence of effector, the HCB suggests that the trait, especially if being obtainable by different means, some escaping regulation, should be considered.

### A (2) off-target modifications

**Fig. 6:**
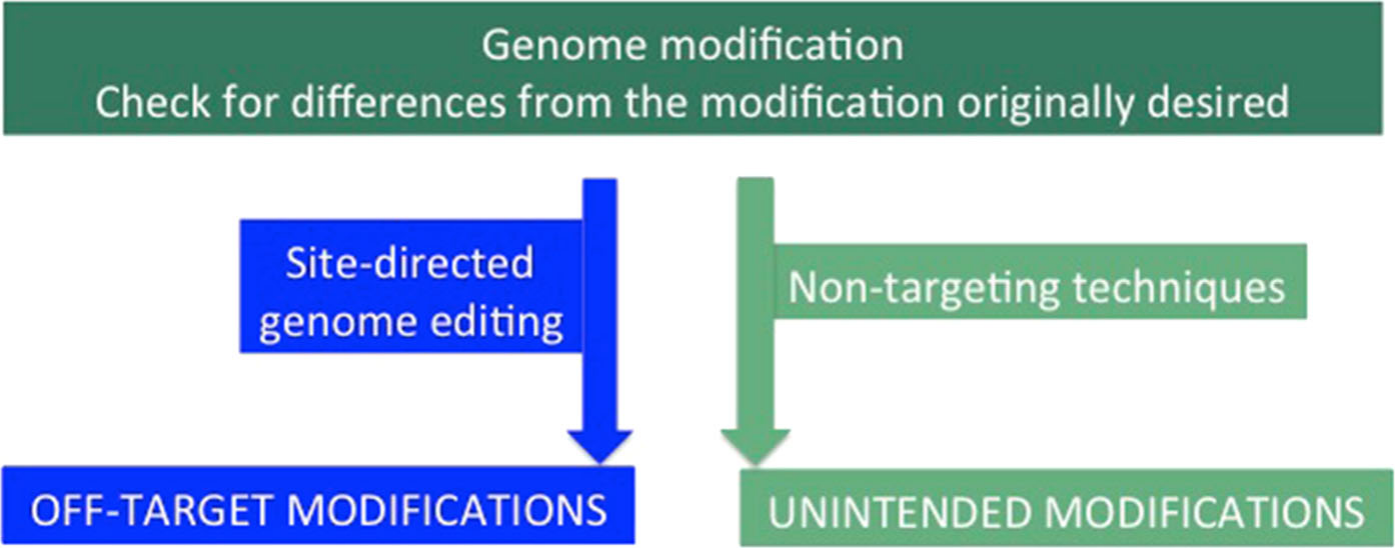
Off-target and unintended modifications. The genomic modification not corresponding to the one expected, are unintended modifications in the case of non-targeting techniques and off-target cuts/modifications, in the case of genome editing, they essentially differ in number

Genome editing carries a potential to trigger genome modification other than the modification originally desired. In case of conventional mutagenesis, such unintended modifications could be massive, whereas for site-directed genome edition they are restricted to the so-called off-target (Fig. 6). Importantly, due to the mode of action of the enzymes, if an off-target modification occurs, it does not necessarily mean that it triggers a phenotypic effect. Since the mechanism supporting off-target cuts is understood, most off-target cuts could be anticipated.

**Fig. 7:**
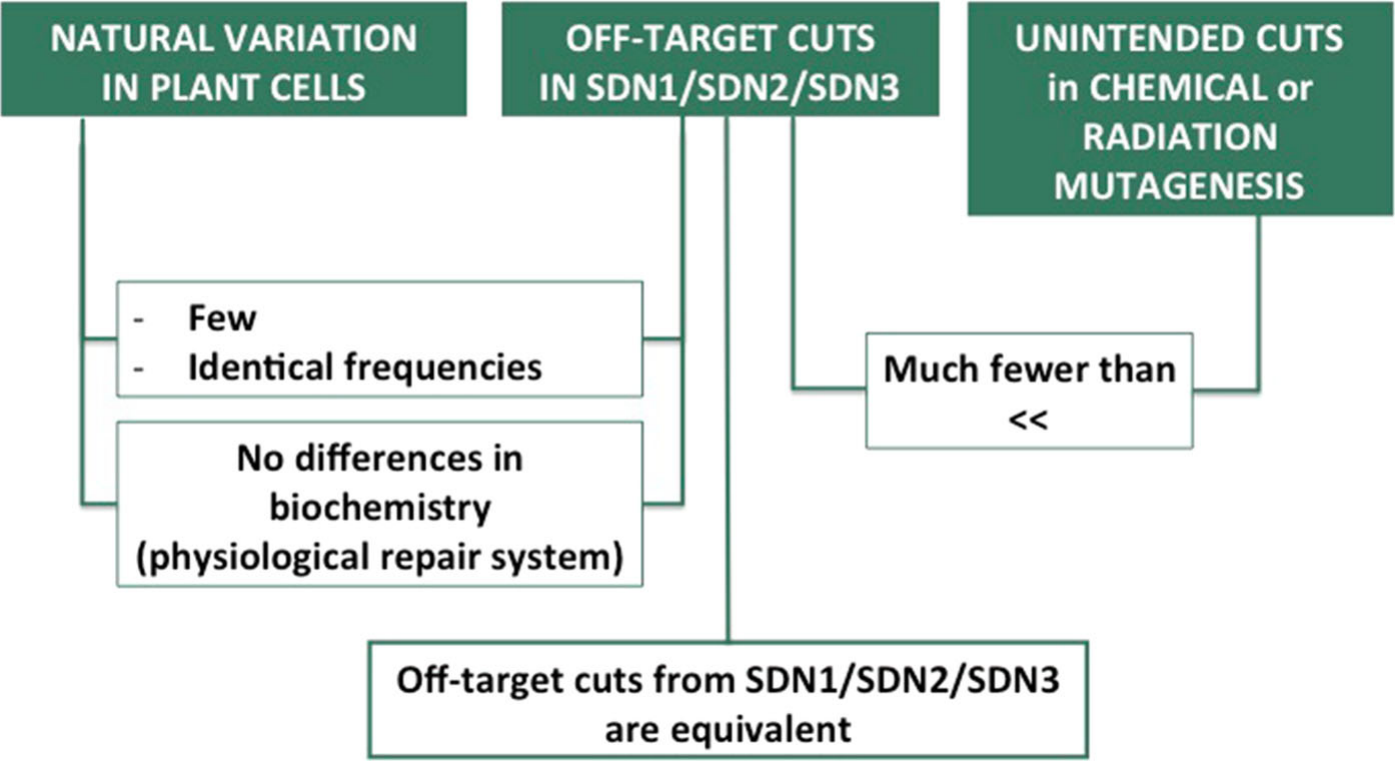
Schematic comparison between genomic natural variation, off-target modifications in SDN1/2/3 genome edition, and unintended modifications from chemical or radiation mutagenesis

It is also important to envision modification in the context of natural genetic variation. To give an order of idea, the rate of spontaneous mutation is theoretically 1 mutation for approximately 100 million base pairs (which is almost corresponding to the genomic size of one seed) per generation in the model plant *A. thaliana* (Lynch 2010) and roughly 120 mutations per seed in wheat. Natural variation in plant cells is of the same order of magnitude and shares biochemical features with off-target modifications in SDN1/SDN2/SDN3 (Fig. 7). Moreover, off-target cuts in SDN2/SDN3 are expected to have few consequences, since the probability that the template DNA recombines within a divergent sequence at the DNA-break site is very small.

Obviously, off-target cuts in SDN1/SDN2/SDN3 are much fewer than the unintended modifications observed in chemical or radiation mutagenesis. Accordingly, the potential off-target cuts will be accessible to characterization and rapid elimination in the breeding process, not to mention technological improvement.

From a technical point of view, off-target cuts are observed when gRNAs are not specific enough and if the nuclease activity (e.g. Cas9) is stably expressed. Recent data show that off-target cuts are limited to a restricted number of sites, those sharing homology with the target of the guide RNA (up to 5 mismatches). Consequently, putative off-target regions may be predicted in silico in known genomes. Identification of off-target genomic modifications is therefore possible by sequencing the in silico predicted off-target potential regions in known genomes, or by whole-genome sequencing. However, the size and diversity of sequence repeats in some genomes, as well as the fact that reference sequences will not necessarily match the sequence of the variety under consideration may limit the identification of off-target cuts. It is also more difficult to identify off-target cuts in plants with large genomes. More importantly, off-target modifications remain not easily distinguishable from the natural mutations found in plants.

As in conventional mutagenesis, it is possible to eliminate off-target cuts, especially if a phenotype is observed, by backcross during the selection process. Of note, it may be technically difficult to eliminate off-target cuts for perennials such as fruit trees or for plants that reproduce mainly through vegetative propagation. In these cases, characterization of the phenotype will constitute the main criteria for release.

Preventing off-target modifications is accessible by an optimal choice and dosage of Cas9 nuclease expression and through the design of highly specific gRNA. Optimization of these parameters reduces the risk of generating off-target modifications. Bioinformatics tools are evolving towards a high specificity gRNA design, and absence of off-target modifications have already been reported in various models including zebrafish, mice, chicken, *Arabidopsis*, and rice. Genetically modified Cas nucleases with high specificity are also available, as well as guidelines for minimizing CRISPR/Cas off-target effects. A good knowledge of plants’ genome sequence is hence a prerequisite. The Scientific Committee of the HCB has discussed the possibility to collect data from sequencing of putative off-target regions identified in silico in routine, for a few years.

### Cell and plant phenotype: Risks associated with the trait: No additional putative risks specific to genome editing compared to other plant breeding techniques

At the level of the trait, no specific and exclusive unintended effects related to genome editing technique have been identified, as compared to other plant breeding techniques (Fig. 4). This is explained by the fact that most trait of agronomic interest are present in existing varieties or in wild plants. The objective seeks by genome edition to introduce characterized alleles from one variety into others. Here, the benefit of using genome edition is the rapidity to obtain the desired variety. Importantly, most traits obtained by genome edition could be also obtained by other plant breeding techniques, not covered by GMO regulations such as crossing of natural variants or products from conventional mutagenesis or covered by GMO regulations such as transgenesis. Among the traits of interest, we could expect to observe disease-resistance or herbicide-tolerant plants at high frequency. Consequently, the potential risks associated to a desired trait, are specific to each individual trait and are not affected by the technique used to generate the variety.

The uncertainty of the risk increases with the degree of novelty of a trait. As mentioned, for most traits those risks will not be specific to genome editing techniques. However, we have to maintain simple mechanisms of survey, since, in the future, it could be possible to derive new functions.

### Impact at the level of the field: risks associated with agricultural practice and adoption rate

Two types of unintended effects could be identified, the first relates to multiplex gene editing which is an exclusive feature of genome edition techniques, while transgenesis could also be used, and the other relates to agricultural impacts, in terms of adoption and practice (Fig. 4).

### C (1) unintended effects associated to the combined targeting of modifications (multiplex gene editing) to derive new traits

**Fig. 8:**
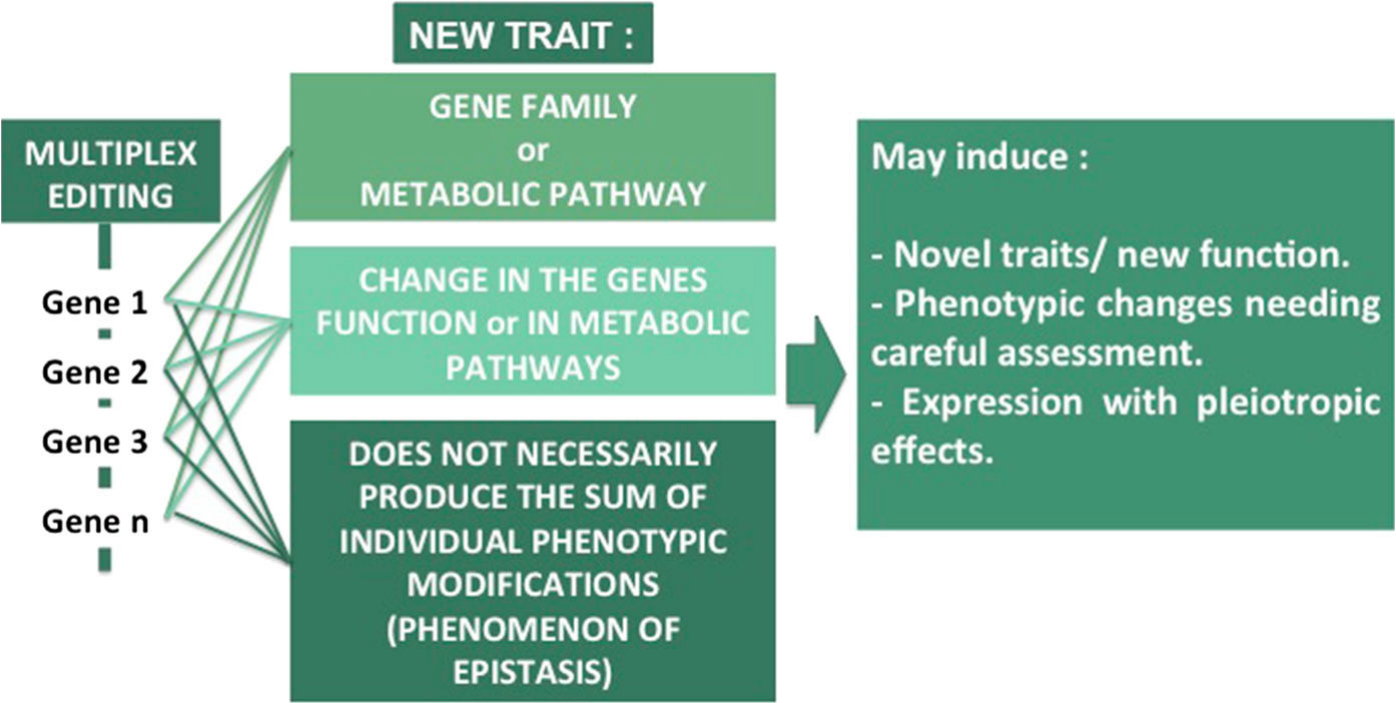
Potential and unintended effects associated with multiplex gene editing

Multiplex gene editing can generate a novel trait in a species by targeting a single gene, a family of genes or a metabolic pathway. By changing the profile of expression of a gene or a metabolic pathway, we do not necessarily produce the sum of each individual phenotype. Also, some modifications could promote epistasis, which stands for the secondary modification of a phenotype. That is why assessment of phenotypic changes in a variety to check for pleiotropic effects could be needed at early stage upon genome edition (Fig. 8).

### C (2) potential acceleration of breeding owing to efficiency and technical ease-of-use of genome editing: related to agricultural practices and ground allocation

The relative efficacy and ease-of-use of genome editing may be beneficial to (1) the direct transfer of a trait of interest identified in a model variety into various other elite varieties of the same species, (2) the translational use of academic knowledge acquired from the understanding of a phenotype in a cultivated or a wild species into other, (3) the selection of variants in up-to-now not modified plants, provided that techniques for genome editing tools are usable in this species and (4) multiplex gene modification of elite varieties.

Such potential acceleration of breeding that may ensue, with a faster pace of production and faster adoption for cultivation of varieties, might affect agronomy. The impact, positive or negative, on ecosystems functioning dynamically deserves attention. In this line, the scientific committee of the HCB suggests a local management with, if necessary, gradual roll-out over time and space, of plants with a novel trait to control the pace of agro-ecosystem change that might result. Additionally, and if needed, the HCB suggests a proportional monitoring in regard to ecological, agro-ecological, economic and societal impacts.

## Conclusion

Having listed the possible unintended events, it appears that, at the molecular level, most question could be addressed at an early step of their development. Hence, product-based characterization remains a relevant approach. Unintended effect is not necessarily synonymous of hazard. However, it is important to keep in mind that a risk is the consequence of both the identified danger/hazard (or source of damage) and the exposure to this danger (likelihood of damage occurring). Exposure varies with the crop adoption. Ideally, potential risks should be evaluated relative to adoption rate. Yet, adoption is often hard to predict, and varies according to social and economic parameters, from the impact of climate to the strategies of the various parties involved in the supply chains. Since exposure relates to the trait rather than the variety and since the same trait can be present in a number of different varieties, adoption of all these varieties must be taken into account for risk assessment. This statement is however not specific to genome edited-plants.

**Fig. 9:**
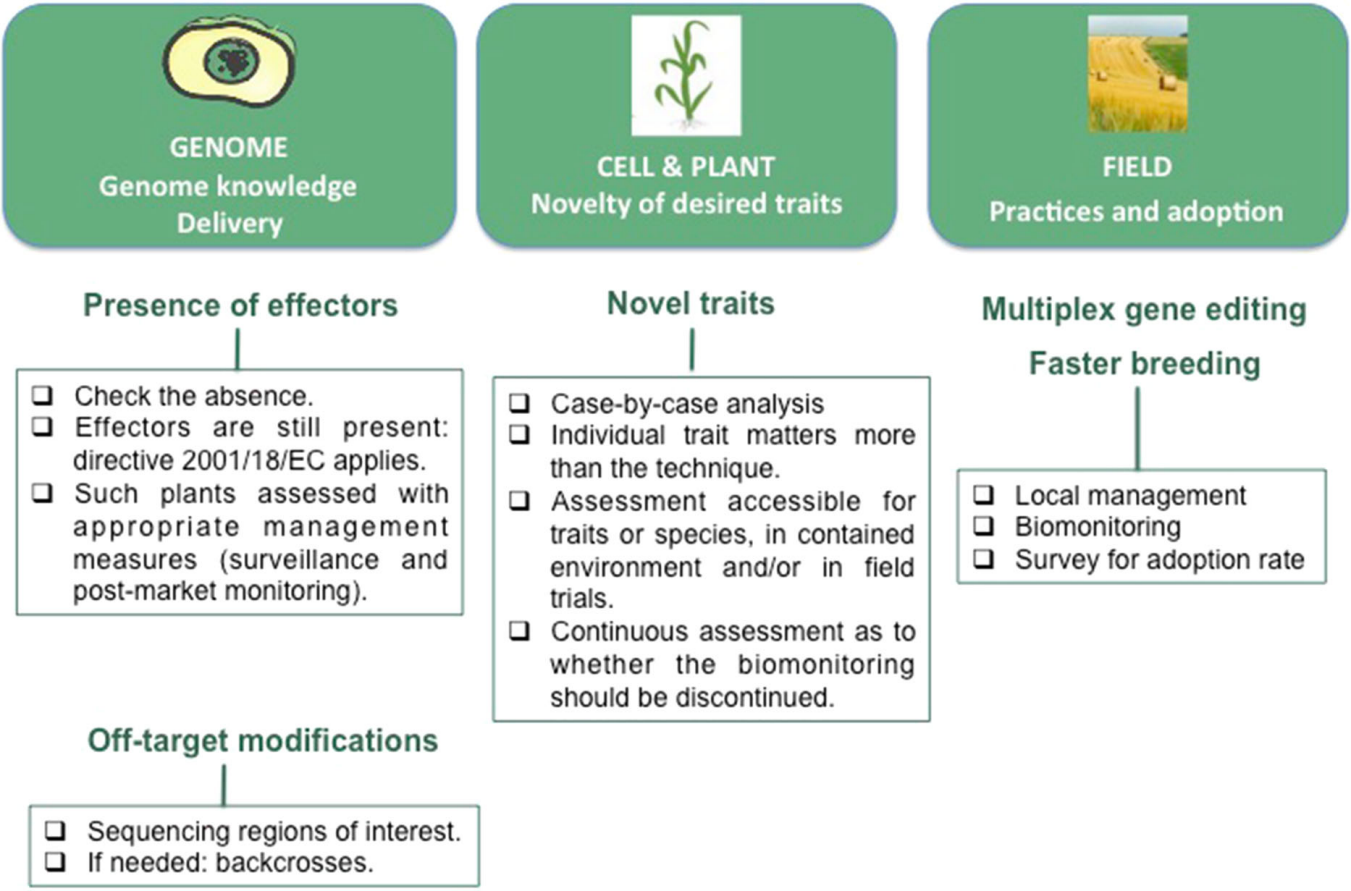
Synthetic view of potential unintended effects and management measures proposed

In proposing to rule genome editing techniques under the directive 2001/18, the CJEU narrows the panel of approaches for evaluation. However, it is possible that the EC or some governments choose to adopt a more proportional scientifically-based approach. For this, case-by-case evaluations have their place, and need optimized protocols such as the one proposed by HCB. To summarize, if risk management measures were to be required related to putative unintended effects, the check-list presented in Fig. 9 may help to cover the relevant questions (Fig. 9).
